# Medical Association of Georgia

**Published:** 1880-05

**Authors:** 


### Editorial.

### MEDICAL ASSOCIATION OF GEORGIA.

This body held its annual meeting in Augusta, on Wednesday, April 21st. The members assembled in Masonic Hall at the hour of eleven, and after prayer by Rev. Dr. Irvine, the address of welcome was delivered by Dr. Henry F. Campbell. The reply was made by Dr. J. T. Johnson, the retiring President, after which the President elect, Dr. Joseph A. Eve, proceeded to deliver his inaugural address. It is said of him that "as he stood before the Association he resembled a great patriarch, whose words of wisdom were eagerly drank in by his loving children."

At the conclusion of the address regular business was commenced by the appointment of Drs. E. L. Connally, E. W. Alfriend and DeSaussure Ford on the Board of Censors, there not being a quorum of the Board present.

The following new members were admitted: Drs. J. W. Barrett, J. E. Allen, W. H. Doughty, Jr., T. R. Wright, C. W. Hickman, A. W. Carswell, J. A. Lane, J. J. Hill, Rev. Robt. Irvine, M.D., and G. C. Dugas.

After a call for papers from the Sections, and the reading by title of those presented, the Association adjourned to 9:30 to-morrow morning.

### SECOND DAY'S PROCEEDINGS.

The Association was called to order by the President, and several letters from absent members were read, and

Dr. W. P. Nicholson, delegate from the Virginia State Medical Society, was introduced.

A protest by physicians of Savannah and Macon against giving the National Board of Health additional powers, was presented and referred to a special committee.

Voluntary papers were read by Drs. DeSaussure Ford, Thos. P. Wright, A. Sibley Campbell and Eugene Foster.

The President announced the following delegates to the American Medical Association at the approaching meeting in New York: Henry Gaither, Thos. R. Wright, Robt. Battey, J. P. Logan, J. B. S. Holmes, H. F. Campbell, W. F. Hitt, E. W. Alfriend, W. J. Howell, T. M. McIntosh, J. C. LeHardy, T. S. Powell, F. A. Stanford, A. G. Whitehead, Thos. Rains, J. W. Bailey, J. G. Thomas, DeSaussure Ford, A. W. Griggs, G. F. Cooper, Wm. O'Daniel, C. H. Hall, K. P. Moore, A. Sibley Campbell and W. B. Wells.

The Committee on Nominations reported the following, who were unanimously elected officers for the ensuing year: for President, Dr. J. C. LeHardy, of Savannah; First Vice President, Dr. E. W. Alfriend, of Albany; Second Vice President, Dr. W. B. Wells, of Red Clay; Censor, Dr. E. L. Connally, of Atlanta.

Committee on Publication—Drs. J. B. Baird, J. T. Johnson, W. S. Armstrong, W. T. Goldsmith and K. P. Moore.

Committee on Necrology — Drs. K. P. Moore, A. S. Campbell, T. S. Hopkins, J. B. S. Holmes and C. B. Leitner.

Orator—Dr. Henry F. Scott, of Atlanta.

The annual address was delivered in the Masonic Hall by Dr. W. S. Kendrick to a large audience of ladies and gentlemen, after which the Association and invited guests repaired to the Planters Hotel, where a sumptuous repast awaited them.

The morning of the third day was occupied in the transaction of unimportant matters, and the Association adjourned to meet in Thomasville on the third Wednesday in April, 1881.

